# Supplementary material for: Methotrexate and Prednisolone compared to placebo and prednisolone in the treatment of severe Erythema Nodosum Leprosum: An international multicentre, double-blind randomised controlled clinical trial—MaPs in ENL
Source: PLoS Negl Trop Dis. 2026 Sep 30;20(9):e0014739. doi: 10.1371/journal.pntd.0014739 (PMC13649114; doi:10.1371/journal.pntd.0014739)
Supplement: Supplementary file 4 [file pntd.0014739.s003.docx]

**S3 File – Adverse Events tables**

Title: Methotrexate and Prednisolone compared to prednisolone alone in Erythema Nodosum Leprosum - an international multicentre, double-blind randomised controlled clinical trial MaPs in ENL

Table 1: Serious treatment related adverse events

| Study Centre | Serious adverse event | Outcome |
| --- | --- | --- |
| TLMI Barabanki Hospital | **Cellulitis with infected ulcer** | **Hospitalised; treated with intravenous antibiotics and surgical debridement; improved and discharged** |
| Bombay Leprosy Project | **Abscess** | **Hospitalised; treated with intravenous antibiotics and surgical drainage; improved and discharged** |
| Bombay Leprosy Project | **Tuberculous meningitis** | **Withdrawn from study; discharged on anti-tuberculosis treatment; recovering at home** |
| Bombay Leprosy Project | **Cellulitis** | **Treated with intravenous antibiotics; improved and discharged; withdrawn from the study** |
| Bombay Leprosy Project | **Pulmonary tuberculosis** | **Treated; improved and discharged; withdrawn from the study** |
