# Supplementary material for: Methotrexate and Prednisolone compared to placebo and prednisolone in the treatment of severe Erythema Nodosum Leprosum: An international multicentre, double-blind randomised controlled clinical trial—MaPs in ENL
Source: PLoS Negl Trop Dis. 2026 Sep 30;20(9):e0014739. doi: 10.1371/journal.pntd.0014739 (PMC13649114; doi:10.1371/journal.pntd.0014739)
Supplement: Supplementary file 7 [file pntd.0014739.s008.docx]

**London, 7 July 2026**

**Epco Hasker**

**Academic Editor**

**PLOS Neglected Tropical Diseases**

**Manuscript ID:** PNTD-D-26-00496

**Title:** ***Methotrexate and Prednisolone compared to placebo and prednisolone in the treatment of Erythema Nodosum Leprosum - an international multicentre, double-blind randomised controlled clinical trial - MaPs in ENL***

Dear Dr Hasker,

We thank you and the reviewers for the careful evaluation of our manuscript and for the constructive comments. We are grateful for the positive assessment of the scientific quality and novelty of our work. We have revised the manuscript accordingly and believe that the changes have strengthened the paper.

All reviewer comments have been addressed individually below. Changes in the revised manuscript are shown using tracked changes. In addition, following the addition of a reference requested by the reviewers, the reference list and all subsequent in-text citation numbers have been updated accordingly. We have also expanded the ethics and trial registration information to include approval by the AHRI/ALERT Ethics Review Committee and registration with the Clinical Trials Registry–India (CTRI/2020/11/029074).

**Response to Reviewer 1**

**1. Comment:** Please state the study hypothesis clearly: that adjunctive methotrexate with prednisolone reduces the risk of, or delays the time to, an ENL flare requiring additional prednisolone compared with prednisolone alone.

**Response:** Thank you for this suggestion. We have revised the Introduction to state the study hypothesis explicitly.

**Changes in manuscript:** We wished to assess the efficacy and safety of weekly oral methotrexate treatment in adults with severe ENL and conducted a double-blind, randomised, placebo-controlled multi-centre trial. We hypothesised that adjunctive methotrexate and prednisolone would reduce the need for additional prednisolone and delay the time to an ENL flare compared with prednisolone alone.

**2. Comment:** The study was underpowered, which limits the ability to draw definitive conclusions. The heterogeneity of the study population, combining acute and recurrent/chronic ENL, also limits the validity of multivariable analyses despite statistical adjustment. Please acknowledge these issues explicitly in the limitations.

**Response:** Thank you for highlighting these important limitations. We have revised the Discussion to acknowledge explicitly that failure to reach the original target sample size reduced statistical power and the precision of the treatment effect estimates. We have also clarified that combining participants with acute and recurrent or chronic ENL introduced clinical heterogeneity and limited our ability to assess whether treatment effects differed according to ENL type, despite prespecified covariate adjustment.

**Changes in manuscript:** We have conducted the largest double-blind, randomised, placebo-controlled trial in ENL to date. Strengths of the study include its multicentre design, the use of clinically meaningful outcome measure with clear criteria for additional prednisolone and the use of validated outcomes measures. The study has important limitations. Recruitment did not reach the original target sample size reducing statistical power. In addition, participants with acute and recurrent or chronic ENL were combined in the primary analysis. Although ENL type was included as a prespecified covariate in the adjusted analysis, clinical heterogeneity between these groups may have resulted in residual heterogeneity and limited our ability to assess whether the treatment effect differed according to ENL type. The final analyses were interpreted with appropriate caution, focusing on the magnitude and direction of effects rather than statistical significance alone.

**3. Comment:** The revision to the statistical analysis plan should be discussed explicitly in the limitations.

**Response:** Thank you for this comment. We agree that the revision to the statistical analysis plan should be acknowledged as a limitation. The SAP was revised before database lock and treatment unblinding to combine participants with recurrent and chronic ENL for the primary analysis. Although this decision was made without knowledge of treatment allocation or outcomes, we acknowledge that the change from the original analysis plan represents a limitation and have added this to the Discussion.

**Changes in manuscript:** The limitations section of the Discussion has been revised to acknowledge the revision to the statistical analysis plan and clarify that this occurred before database lock and treatment unblinding.

**4. Comment:** The resolution and clarity of the figures should be improved.

**Response:** Thank you. We have prepared the figures as separate high-resolution files in accordance with PLOS figure requirements and will upload them individually at resubmission. We have also checked the figure legends and corrected the missing/duplicated legends.

**Changes in manuscript:** No changes made.

**5. Comment:** Given the study limitations, the conclusions should be less definitive and should state that there was no evidence of benefit, rather than making an absolute conclusion that methotrexate is ineffective. The limitations should be explicitly discussed in the manuscript, including the Discussion and Abstract.

**Response:** Thank you for this comment. We agree that the conclusions should be interpreted in the context of the limitations of the study. We have already expanded the Discussion to emphasise the reduced sample size, the revised statistical analysis plan and the heterogeneity of the study population. We believe the conclusions are appropriately balanced and accurately reflect the findings of the trial. We therefore have not made further changes to the wording of the conclusions.

**Changes in manuscript:** No further changes made.

**6. Comment:** The higher representation of participants from one location and the resulting limitations in representativeness and generalisability should be discussed.

**Response:** Thank you for highlighting this limitation. We have revised the Discussion to acknowledge the imbalance in recruitment between participating countries and its implications for representativeness and generalisability. Although the trial was conducted across multiple countries, most participants were recruited in India. We have therefore clarified that the findings predominantly reflect the study population recruited in India and may not be fully generalisable to individuals with severe ENL in other geographical and healthcare settings.

**Changes in manuscript:** The limitations paragraph in the Discussion has been revised to acknowledge the geographical imbalance in recruitment and its implications for the representativeness and generalisability of the findings.

**Response to Reviewer 2**

**1. Comment:** Please consider changing the title to specify that the trial concerns severe ENL.

**Response:** Thank you for this suggestion. We agree that specifying the severity of ENL in the title more accurately reflects the study population. We have therefore revised the title to specify that the trial was conducted in individuals with severe ENL.

**Changes in manuscript: “Methotrexate and prednisolone for the treatment of severe Erythema Nodosum Leprosum (MaPs in ENL): a double-blind, randomised, placebo-controlled multicentre trial.”**

**2. Comment:** For severe ENL after completion of multidrug therapy, prednisolone plus clofazimine is recommended treatment. Please explain why this was not used as the reference treatment in the trial.

**Response:** Thank you for this comment. We respectfully note that prednisolone combined with clofazimine is not recommended as the standard reference treatment for severe ENL after completion of multidrug therapy(1). Evidence supporting clofazimine for the treatment of ENL is limited, and its slow onset of action makes it unsuitable for the rapid control of acute severe ENL inflammation. Systemic corticosteroids remain the principal treatment for severe ENL, and prednisolone was therefore used as the standard background treatment in both trial arms. The trial was designed to determine whether the addition of methotrexate could improve ENL control and reduce corticosteroid requirements compared with prednisolone alone. We therefore have not made changes to the manuscript in response to this comment.

**Changes in manuscript:** No changes made.

**Response to Reviewer 3**

**Abstract**

**1. Comment:** Include the dosage and duration of the study drugs.

**Response:** Thank you for this comment. We have revised the Abstract Methods section to specify the methotrexate dose and duration and the initial prednisolone dose and treatment duration.

**Changes in manuscript:** Adults aged 18–60 years with severe ENL were randomised to receive oral methotrexate (15mg, increased to 20mg in participants weighing ≥ 60kg) for 48 weeks and prednisolone, or matching placebo and prednisolone. All participants received an identical 20-week prednisolone regimen, starting at 40mg daily, and were followed for 60 weeks.

**Introduction**

**2. Comment:** Line 100: change “Individuals” to lower case.

**Response:** Thank you. This has been corrected as suggested.

**Changes in manuscript:** “Individuals” has been changed to lower case.

**3. Comment:** Lines 100–102: add a reference to the statement describing the proportion of individuals with LL and BL leprosy affected by ENL.

**Response:** Thank you. We have added an appropriate reference to support the reported proportion of individuals with lepromatous (LL) and borderline lepromatous (BL) leprosy who develop ENL.

**Changes in manuscript:** A supporting reference has been added to the relevant statement in the Introduction.

**4. Comment:** Line 114: correct the grammar of the sentence describing WHO recommendations for corticosteroid treatment.

**Response:** Thank you. The sentence describing the WHO recommendation for corticosteroid treatment of severe ENL has been revised for grammatical clarity.

**Changes in manuscript:** ENL is often chronic requiring prolonged courses of high dose oral corticosteroid therapy which WHO recommends as first-line treatment.

**5. Comment:** Line 115: add two or three examples of adverse effects associated with corticosteroid treatment.

**Response:** Thank you for this suggestion. We have revised the Introduction to provide examples of important adverse effects associated with prolonged corticosteroid therapy.

**Changes in manuscript:** Corticosteroid therapy in patients with ENL is associated with adverse effects, including diabetes mellitus, hypertension and osteoporosis, and significant mortality in some settings**.**

**6. Comment:** Lines 131–134: briefly report the clinical outcome of the previous methotrexate trial conducted in Bangladesh, rather than reporting only the absence of serious adverse events.

**Response:** Thank you for this suggestion. We have revised the Introduction to report the clinical outcomes of the previous trial. We now clarify that, although ENL severity improved during treatment in both groups, there was no significant difference in reaction severity between the methotrexate plus prednisolone and prednisolone monotherapy groups, and no significant corticosteroid-sparing effect was demonstrated. We have also retained the relevant context regarding the small sample size and short study duration.

**Changes in manuscript:** The Introduction has been revised to summarise the clinical findings of the previous trial, including the absence of a significant between-group difference in reaction severity and the lack of a demonstrated corticosteroid-sparing effect.

**7. Comment:** Line 138: use the abbreviation HRQoL, as health-related quality of life has already been defined.

**Response:** Thank you. We have replaced “health-related quality of life” with the previously defined abbreviation “HRQoL”, as suggested.

**Changes in manuscript:** “Health-related quality of life” has been replaced with “HRQoL”.

**8. Comment:** Line 140: replace “We wished” with “We aimed” and include the duration of methotrexate treatment.

**Response:** Thank you. We have replaced “We wished” with “We aimed” and specified the 48-week duration of methotrexate treatment.

**Changes in manuscript:** The final paragraph of the Introduction has been revised to state the study aim and specify that methotrexate was administered for 48 weeks.

**Methods**

**9. Comment:** Reference 29 is incomplete; add the journal details.

**Response:** Thank you. We have corrected Reference 29 by adding the missing journal details.

**Changes in manuscript:** Reference 29 has been amended to include the complete journal citation details.

**10. Comment:** Line 168: complete the sentence defining ENL, which currently ends with “tender papular and/or nodular skin”.

**Response:** Thank you. The sentence defining ENL has been completed by adding the missing word “lesions”.

**Changes in manuscript:** The definition of ENL has been corrected to read “tender papular and/or nodular skin lesions”.

**11. Comment:** Line 169: include the definitions of acute, recurrent and chronic ENL.

**Response:** Thank you. We have added the definitions used to classify acute, recurrent and chronic ENL in the trial.

**Changes in manuscript:** The Methods section has been revised to include the definitions of acute, recurrent and chronic ENL used for participant classification.

**12. Comment:** Line 183: identify the specialist company that prepared the methotrexate and placebo tablets.

**Response:** Thank you. We have revised the Methods to identify the companies responsible for the study medications. Methotrexate was manufactured by Cipla, and the matching placebo tablets were procured by Bilcare.

**Changes in manuscript:** The Methods section has been revised to identify Cipla as the manufacturer of methotrexate and Bilcare as the company that procured the matching placebo tablets.

**13. Comment:** Line 193: provide a reference or further methodological information for the validated high-performance liquid chromatography method and state where the testing was conducted.

**Response:** Thank you for this comment. The HPLC method was a validated analytical method used by the testing laboratory and does not have a specific published reference. We have revised the Methods to provide further methodological information and to identify the laboratory where the analyses were conducted.

**Changes in manuscript:** The Methods section now states that drug quality testing was conducted at the LSHTM Bioanalytical Laboratory using a validated high-performance liquid chromatography method under Good Laboratory Practice standards.

**14. Comment:** Line 202: state clearly how many weeks methotrexate was administered.

**Response:** Thank you. We have revised the Methods to state explicitly that methotrexate was administered at the treatment dose for 48 weeks, followed by a 4-week dose-tapering period.

**Changes in manuscript:** Participants in the intervention arm received weekly oral methotrexate at the treatment dose for 48 weeks, followed by 4-week dose taper.

**15. Comment:** Line 205: explain why the methotrexate dose was increased to 20 mg after two months in participants weighing ≥60 kg.

**Response:** Thank you for this comment. The dose escalation to 20 mg weekly in participants weighing ≥60 kg was incorporated into the protocol to ensure an adequate therapeutic dose in participants with higher body weight. The doses used in the trial were within the established therapeutic range for low-dose methotrexate in inflammatory diseases (7.5–25 mg weekly). The increase from 15 mg to 20 mg was introduced after the initial treatment period to allow assessment of tolerability and safety before dose escalation. The dosing regimen was prespecified in the published trial protocol.

**Changes in manuscript:** No changes made.

**16. Comment:** Line 205 and Table 1: resolve the inconsistency between dose 9 and week 8 for the increase to 20 mg methotrexate.

**Response:** Thank you for this comment. We reviewed the treatment schedule and confirmed that there is no inconsistency between the text and Table 1. The first methotrexate dose was administered at enrolment (week 0); therefore, dose 9 was administered at week 8. Thus, “dose 9 onwards” in the text and “week 8” in Table 1 refer to the same point in the treatment schedule.

**Changes in manuscript:** No changes made.

**17. Comment:** Table 1: consider simplifying the table by combining rows with identical dosing, for example weeks 2–3 and weeks 4–5.

**Response:** Thank you for this suggestion. We considered combining rows with identical doses; however, we have retained the weekly presentation because it provides a clear representation of the complete treatment schedule and facilitates interpretation of the timing of dose changes for both methotrexate and prednisolone.

**Changes in manuscript:** No changes made.

**18. Comment:** Line 220: clarify whether folic acid was omitted on the weekly methotrexate/placebo dosing day.

**Response:** Thank you for this comment. Folic acid was omitted on the day of weekly methotrexate or placebo administration. We have clarified this in the Methods.

**Changes in manuscript:** The Methods section has been revised to state that folic acid was administered daily, except on the day of weekly methotrexate or placebo administration.

**19. Comment:** Line 290: add the target number of participants per trial arm to the sample-size description.

**Response:** Thank you. We have clarified the sample-size description by stating the target number of participants per trial arm.

**Changes in manuscript:** The sample-size section now specifies that the target sample size was 416 participants, with 208 participants per trial arm.

**20. Comment:** Lines 304–306: specify the countries in which civil unrest affected recruitment.

**Response:** Thank you for this suggestion. We have retained the description of civil unrest without identifying the affected country. The purpose of this statement is to explain the operational challenges that affected recruitment rather than to attribute these challenges to a particular study setting. We considered that identifying individual countries would not contribute to the interpretation of the trial findings and could unnecessarily associate specific study sites with political circumstances outside the scope of the study.

**Changes in manuscript:** No changes made.

**21. Comment:** Lines 325–326: explain whether country of origin and bacterial index were considered as covariates in the Cox models. Please also address whether the association between the number/extent of nodules and ENL severity reported in the Brazilian study (PMID: 32358972) was observed in the MaPs data.

**Response:** Thank you for this comment. Country and bacterial index were not included as covariates in the prespecified Cox proportional hazards models. The adjustment variables were ENL type (acute versus recurrent/chronic), sex, age category, weight, EESS score at enrolment and WHO-MDT status, as stated in the Methods.

The relationship between the clinical components of the EESS, including cutaneous lesion burden, and overall ENL severity has been evaluated separately in a longitudinal analysis of the MaPs cohort. This analysis was conducted using the cohort as a whole, rather than by treatment arm, as the main trial analyses showed no evidence of differences in clinical outcomes between treatment groups. The results of this longitudinal EESS analysis are currently being prepared for publication.

**Changes in manuscript:** No changes made.

**22. Comment:** State whether the trial was reported according to the CONSORT guidelines.

**Response:** Thank you. We have clarified that the trial was reported in accordance with the CONSORT guidelines.

**Changes in manuscript:** The Methods section has been revised to state that the trial was reported in accordance with the CONSORT guidelines.

**23. Comment:** Consider combining the trial design/conduct and trial oversight and/or ethics sections.

**Response:** Thank you for this suggestion. We have retained the trial design and conduct and trial oversight sections separately, as they describe distinct aspects of the study. The trial design and conduct section describes the study framework, sponsorship and conduct, whereas the trial oversight section provides information on ethical approvals, informed consent and independent safety oversight.

**Changes in manuscript:** No changes made.

**Results**

**24. Comment:** Line 353: provide an overview, including a flow diagram, showing the 94 screened individuals who were excluded and the reasons for exclusion.

**Response:** Thank you for this comment. The 94 individuals who were screened but not randomised have been evaluated as a separate cohort, including detailed analysis of the reasons for exclusion and their clinical characteristics and outcomes. These findings form a separate manuscript that will be submitted for publication following publication of the main trial results. We therefore have not included this detailed analysis in the present manuscript, which reports the results of the randomised trial population. The existing CONSORT flow diagram reports the total numbers screened, excluded and randomised.

**Changes in manuscript:** No changes made.

**25. Comment:** Line 355: add percentages by country to show the imbalance in recruitment more clearly.

**Response:** Thank you for this suggestion. We have retained the presentation of recruitment by study centre as absolute numbers rather than adding percentages by country. The trial was designed and analysed as a multicentre study with a pooled study population, rather than to provide country-specific estimates or comparisons. We considered that adding country-level percentages would not contribute to interpretation of the prespecified trial analyses. The imbalance in recruitment across study centres is already reported and has been explicitly acknowledged as a limitation affecting representativeness and generalisability.

**Changes in manuscript:** No changes made.

**25. Comment:** Table 2: clarify the definitions of “recent MDT” and “long-term MDT”.

**Response:** Thank you for this comment. The definitions are already provided directly in Table 2: recent MDT is defined as completion of MDT less than 3 years before enrolment, and long-term MDT as completion of MDT 3 or more years before enrolment.

**Changes in manuscript:** No changes made.

**26. Comment:** Table 2 and text: report IQR consistently, using the 25th and 75th percentiles rather than a single number in the table.

**Response:** Thank you for this comment. We have revised the presentation of continuous variables summarised using the median and IQR to report the 25th and 75th percentiles consistently in the text and Table 2.

**Changes in manuscript:** Table 2 and the corresponding Results text have been revised to report median values with the 25th and 75th percentiles (Q1–Q3), rather than reporting the IQR as a single value.

**27. Comment:** Line 625: clarify that the statement refers to the previous trial conducted in Bangladesh and add the reference.

**Response:** Thank you for this comment. We have clarified that this statement refers to the previous randomised trial of methotrexate for ENL conducted in Bangladesh and have added the corresponding reference.

**Changes in manuscript:** The Discussion has been revised to specify that the statement refers to the previous trial conducted in Bangladesh, and the relevant reference has been added.

**General comments**

**28. Comment:** Although this was a multinational trial, 85% of participants were recruited in India. Please report whether primary and secondary outcomes differed between India and the other three countries, and discuss the implications of the recruitment imbalance for interpretation and generalisability. Please consider this in relation to the positive signal reported in the small French study by Jaume et al. (PLOS Neglected Tropical Diseases, 2023).

**Response:** Thank you for this comment. The trial was designed and analysed as a multicentre study and was not powered to assess geographical heterogeneity in treatment effects. Given the small number of participants recruited outside India, comparisons of primary and secondary outcomes between India and the other participating countries would have very limited statistical power and could produce unreliable estimates. We therefore did not undertake additional post hoc subgroup analyses by geographical location.

We agree that the predominance of recruitment from India limits the geographical representativeness of the trial population and have strengthened the Discussion accordingly. We have also expanded the existing discussion of the French multicentre retrospective cohort reported by Jaume et al.. Although this study reported favourable outcomes with methotrexate, only six participants had ENL, and differences in study design, population and outcome assessment limit direct comparison with the present randomised trial. We acknowledge that geographical variation in treatment response cannot be excluded.

**Changes in manuscript:** The Discussion has been revised to expand the comparison with the retrospective French cohort reported by Jaume et al. and to acknowledge that the predominance of recruitment from India limits geographical representativeness and that geographical variation in treatment response cannot be excluded.

**29. Comment:** Given the initial prednisolone dose of 40 mg and the number of methotrexate/placebo tablets, particularly for participants weighing >60 kg who received 20 mg methotrexate/placebo, was pill burden a concern for participants? Please discuss.

**Response:** Thank you for this comment. Pill burden was not formally assessed as an outcome in the trial, and therefore we are unable to evaluate its acceptability or impact directly. Methotrexate or placebo was administered once weekly, whereas prednisolone was administered daily according to the tapering regimen. Although participants receiving 20 mg methotrexate or placebo took eight tablets on the weekly dosing day, no concerns regarding pill burden were systematically collected. We have therefore not added discussion of this issue to the manuscript, as this would be speculative.

**Changes in manuscript:** No changes made.

**30. Comment:** Based on the available data, is there a reason to study methotrexate further in ENL? Please elaborate and include a brief discussion in the manuscript.

**Response:** Thank you for this important comment. We agree that the implications of the findings for further research should be stated explicitly. The Discussion already notes that the observed differences between treatment groups were small and that a clinically meaningful benefit is unlikely to have been demonstrated with a larger sample size. We have now clarified that the findings do not support further trials of methotrexate using similar doses and treatment regimens in severe ENL**.**

**Changes in manuscript:** The Discussion has been revised to state: “Our findings do not support further trials of methotrexate at similar doses and treatment regimens for severe ENL.”

**Journal requirements and technical checks**

**1. Comment:** Upload all main figures as separate .tif or .eps files.

**Response:** All main figures have been prepared and uploaded as separate high-resolution .tif files in accordance with the journal requirements.

**Changes in manuscript:** No changes to the manuscript. Revised figure files have been uploaded separately.

**2. Comment:** Add a complete list of Supporting Information legends after the reference list.

**Response:** Thank you. A complete list of Supporting Information legends is already provided after the reference list in the manuscript.

**Changes in manuscript:** No changes to the manuscript.

**3. Comment:** Amend the Financial Disclosure statement to identify, by initials, the authors who received each grant.

**Response:** The Financial Disclosure statement has been amended accordingly.

**Changes in manuscript:** The Financial Disclosure statement has been amended accordingly.

**4. Comment:** Add the missing Figure 4 legend and remove the duplicated Figure 2 legend.

**Response:** Thank you. We have checked the figure legends and confirm that the Figure 4 legend is present and that there is no duplicated Figure 2 legend in the revised manuscript.

**Changes in manuscript:** No changes made.

**5. Comment:** Confirm the complete funding information, including grant ITCRZM25, the correct order of funding contributions, and the statement describing the funders’ role.

**Response:** Thank you. We have reviewed and revised the funding information to include grant ITCRZM25, present the funding contributions in the correct order, and include the required statement describing the role of the funders.

**Changes in manuscript:** The Financial Disclosure statement has been revised to include the complete funding information, including grant ITCRZM25, the correct order of funding contributions, and the statement describing the role of the funders.

**6. Comment:** Check the journal-amended Competing Interests statement and confirm that it is correct.

**Response:** Thank you. We have reviewed the journal-amended Competing Interests statement and confirm that it is correct.

**Changes in manuscript:** No changes to the manuscript.

We thank the Academic Editor and reviewers again for their careful consideration of our manuscript and constructive comments. We hope that the revised manuscript is now suitable for publication in *PLOS Neglected Tropical Diseases*.

Yours sincerely,

**Barbara de Barros**

On behalf of all authors
