# Supplementary material for: Methotrexate and Prednisolone compared to placebo and prednisolone in the treatment of severe Erythema Nodosum Leprosum: An international multicentre, double-blind randomised controlled clinical trial—MaPs in ENL
Source: PLoS Negl Trop Dis. 2026 Sep 30;20(9):e0014739. doi: 10.1371/journal.pntd.0014739 (PMC13649114; doi:10.1371/journal.pntd.0014739)
Supplement: Peer review history

# Author comment: Author response to Decision Letter 0

**DOI:** 10.1371/journal.pntd.0014739.r001

**Transfer Alert**

This paper was transferred from another journal. As a result, its full editorial history (including decision letters, peer reviews and author responses) may not be present.

16 Mar 2026

# Peer review: Decision Letter 0

**DOI:** 10.1371/journal.pntd.0014739.r002

**Authors:** Epco Hasker, Ana LTO Nascimento

3 Jul 2026

PNTD-D-26-00496

Methotrexate and Prednisolone compared to placebo and prednisolone in the treatment of Erythema Nodosum Leprosum - an international multicentre, double-blind randomised controlled clinical trial - MaPs in ENL

PLOS Neglected Tropical Diseases

Dear Dr. Barros,

Thank you for submitting your manuscript to PLOS Neglected Tropical Diseases. After careful consideration, we feel that it has merit but does not fully meet PLOS Neglected Tropical Diseases's publication criteria as it currently stands. Therefore, we invite you to submit a revised version of the manuscript that addresses the points raised during the review process.

Please submit your revised manuscript within by Aug 31 2026 11:59PM. If you will need more time than this to complete your revisions, please reply to this message or contact the journal office at plosntds@plos.org. When you're ready to submit your revision, log on to https://www.editorialmanager.com/pntd/ and select the 'Submissions Needing Revision' folder to locate your manuscript file.

Please include the following items when submitting your revised manuscript:

* A letter that responds to each point raised by the editor and reviewer(s). You should upload this letter as a separate file labeled 'Response to Reviewers'. This file does not need to include responses to any formatting updates and technical items listed in the 'Journal Requirements' section below.

* A marked-up copy of your manuscript that highlights changes made to the original version. You should upload this as a separate file labeled 'Revised Manuscript with Track Changes'.

* An unmarked version of your revised paper without tracked changes. You should upload this as a separate file labeled 'Manuscript'.

If you would like to make changes to your financial disclosure, competing interests statement, or data availability statement, please make these updates within the submission form at the time of resubmission. Guidelines for resubmitting your figure files are available below the reviewer comments at the end of this letter.

As the corresponding author, your ORCID iD is verified in the submission system and will appear in the published article. PLOS supports the use of ORCID, and we encourage all coauthors to register for an ORCID iD and use it as well. Please encourage your coauthors to verify their ORCID iD within the submission system before final acceptance, as unverified ORCID iDs will not appear in the published article. Only the individual author can complete the verification step; PLOS staff cannot verify ORCID iDs on behalf of authors.

We look forward to receiving your revised manuscript.

Kind regards,

Epco Hasker

Academic Editor

PLOS Neglected Tropical Diseases

Ana LTO Nascimento

Section Editor

PLOS Neglected Tropical Diseases

Shaden Kamhawi

co-Editor-in-Chief

PLOS Neglected Tropical Diseases

orcid.org/0000-0003-4304-636XX

Paul Brindley

co-Editor-in-Chief

PLOS Neglected Tropical Diseases

orcid.org/0000-0003-1765-0002

**Additional Editor Comments (if provided):**

**Journal Requirements:**

If the reviewer comments include a recommendation to cite specific previously published works, please review and evaluate these publications to determine whether they are relevant and should be cited. There is no requirement to cite these works unless the editor has indicated otherwise.

1) Please upload all main figures as separate Figure files in .tif or .eps format. For more information about how to convert and format your figure files please see our guidelines:

https://journals.plos.org/plosntds/s/figures

2) We have noticed that you have uploaded Supporting Information files, but you have not included a list of legends. Please add a full list of legends for your Supporting Information files after the references list.

3) Please amend your detailed Financial Disclosure statement. This is published with the article. It must therefore be completed in full sentences and contain the exact wording you wish to be published.

i) State the initials, alongside each funding source, of each author to receive each grant. For example: "This work was supported by the National Institutes of Health (####### to AM; ###### to CJ) and the National Science Foundation (###### to AM).".

If you did not receive any funding for this study, please simply state: u201cThe authors received no specific funding for this work.u201d

4) We have noticed that you have uploaded Figure files, but you have not included a legend for Figure 4 and also Figure 2 legend was repeated twice. Please add a full list of legends for your Supporting Information files after the references list.

5) Your current Financial Disclosure states, "This study was funded by The Hospital and Homes of St Giles (grant number ITCRZM25) and the Leprosy Research Initiative, supported by the Turing Foundation and plan (grant number LRI 704.16.71). The funders had no role in study design, data collection and analysis, decision to publish, or preparation of the manuscript.". However, your funding information on the submission form not indicates grant number ITCRZM25. Please indicate by return email the full and correct funding information for your study and confirm the order in which funding contributions should appear. Please be sure to indicate whether the funders played any role in the study design, data collection and analysis, decision to publish, or preparation of the manuscript.

6) We have amended your Competing Interest statement to comply with journal style. We kindly ask that you double check the statement and let us know if anything is incorrect.

**Reviewers' Comments:**

Reviewer's Responses to Questions

**Key Review Criteria Required for Acceptance?**

As you describe the new analyses required for acceptance, please consider the following:

**Methods**

-Are the objectives of the study clearly articulated with a clear testable hypothesis stated?

-Is the study design appropriate to address the stated objectives?

-Is the population clearly described and appropriate for the hypothesis being tested?

-Is the sample size sufficient to ensure adequate power to address the hypothesis being tested?

-Were correct statistical analysis used to support conclusions?

-Are there concerns about ethical or regulatory requirements being met?

Reviewer #1: The study objectivies are well articulated and the study design is appropriate.

It would be beneficial if the hypothesis is clearly stated - that adjunctive methotrexate with prednisolone reduces the risk or delays time to ENL flare ( requiring additional prednisolone) compared with prednisolone slone.

The study population is clearly defined, however, underpowered sample size remains a major limitation and this further limits the ability to make definitive conclusions from the study. The heterogeneity of the sample - combining acute and chronic ENL populations also limits the validity of multivariable analysis despite statistical adjustments. This needs to be acknowleged in the limitations as well.

Reviewer #2: Really excellent article, following state of the art set-up and methodology

Reviewer #3: R150: ref 29 not complete, journal not included.

R168: … skin lesions (sentence incomplete).

R169: Include under definitions, the definitions of acute, recurrent and chronic ENL.

R183: ‘… a specialist company.’ This sounds a bit vague, perhaps include the name of the pharmaceutical company involved?

R193: ‘… validated high performance liquid chromatography’ add reference to how this was conducted if possible and where was this conducted?

R202: Under ‘Trial Intervention’ it would be helpful to include a clear sentence how long MTX was given (number of weeks) as this hasn’t been mentioned anywhere until this section.

R205: why was MTX increased to 20mg after only 2 months in individuals >60kg?

R205: in the text this increase in dose is mentioned to occur at dose 9 while in Table 1 it is mentioned from week 8. This inconsistency in reporting may unnecessary lead to confusion.

R215: Table 1 could also be simplified by combining rows, eg week 2+3, week 4+5 etc

R220: clarify that (I presume) folic acid was skipped on the weekly MTX day.

R290: Sample size – add in brackets how many individuals per arm targeted

R304-6: Civil unrest in which countries? Please be more specific

R325-6: The covariates included in the Cox models were selected a priori. Did the authors consider to include country of origin or bacillary index (BI-) as variables? Although BI is not reported to be associated with ENL severity in the literature, it remains a proxy for bacterial burden and disease phenotype and may still be relevant for adjustment. Similarly, given the multicountry design, country of origin could be an important source of heterogeneity. In addition, a small study from Brazil showed a correlation between the number and extension of nodules and the severity of ENL (PMID: 32358972). Was this seen in the data?

- Was CONSORT guideline used to report the data? Include a statement on this.

- Trial design/conduct and oversight and/or ethics could be combined into one section.

**********

**Results**

-Does the analysis presented match the analysis plan?

-Are the results clearly and completely presented?

-Are the figures (Tables, Images) of sufficient quality for clarity?

Reviewer #1: The results are consistent and transparent with the revised statistical plan. However, the revision is the analysis plan needs to be discussed explicitly in the limitations.

While the tables and figuers are relevant and insightful, the resolution of the images can be refined for better clairty.

Reviewer #2: Results are presented very clearly and fully in line with the analysis plan

Reviewer #3: Results:

R353: 94 screened participants (41%) were excluded from the trial. It would be helpful to provide an overview including a graph illustrating the flow of the trial and including reasons persons were excluded.

R355: add percentages to illustrate more clearly the imbalance in inclusion per country.

Table 2 what is exactly meant with ‘recent MDT’ and ‘longterm MDT’? This should be clarified as currently not clear. Also, please be consistent in reporting IQR, in table only reported with one number (the difference in the width?) while in the text reported as a range 25th and 75th percentile.

R625: clarify a bit more this is referring to the trial from Bangladesh and include reference.

**********

**Conclusions**

-Are the conclusions supported by the data presented?

-Are the limitations of analysis clearly described?

-Do the authors discuss how these data can be helpful to advance our understanding of the topic under study?

-Is public health relevance addressed?

Reviewer #1: The conclusions are consistent with the data presented. However, considering the limitations in the study, it would be beneficial to not be too definitive with the conclusion but rather describe that there was no evidence of benefit. Even definitive conclusions would benefit from stronger argumentation despite it being an underpowered study.

Overall, the limitations could be explicitly discussed.

Reviewer #2: Conclusion is clear and honest

Reviewer #3: see general comments

**********

**Editorial and Data Presentation Modifications?**

Use this section for editorial suggestions as well as relatively minor modifications of existing data that would enhance clarity. If the only modifications needed are minor and/or editorial, you may wish to recommend “Minor Revision” or “Accept”.

Reviewer #1: The manuscript will benefit from minor improvements stating the hypothesis, discussing limitations - both in discussion as well as abstract and improving the image resolutions.

Reviewer #2: No modifications needed

Reviewer #3: Abstract: include dosage and duration of drugs

Introduction:

R100: individuals (no capital)

R100-2: ‘ENL ….. BL leprosy’ – suggest to add reference to this sentence

R114: ‘…which WHO the recommend as first-line treatment ..’ sentence grammatically not correct

R115: add 2 or 3 examples of adverse effects

R131-4: please include very briefly the outcome of the MTX trial conducted in Bangladesh (ref 28) as it seems the current trial was based on these preliminary findings. Currently only SAE are reported, not clinical effect.

R138: health-related QoL can be abbreviated as mentioned earlier in paper

R140: We aimed (instead of wished); I suggest to include duration of MTX treatment here as well

**********

**Summary and General Comments**

Use this section to provide overall comments, discuss strengths/weaknesses of the study, novelty, significance, general execution and scholarship. You may also include additional comments for the author, including concerns about dual publication, research ethics, or publication ethics. If requesting major revision, please articulate the new experiments that are needed.

Reviewer #1: The study has notable strengths when it comes to the design of the RCT, multicentric collaboration and well detailed statistical analysis. The findings, while still not positive towards the hypothesis, still remain highly relevant given the limited treatment options for ENL.

The study is significantly limited by its underpowered smaple size resulting in limitations to draw any definitive conclusions. The heteroginity and higher representation of sample from a particular location over the others also adds to limitations in representativeness. Nevertheless, it remains a highly valuable study. Detailing the limitations and re-defining the conclusions being careful to not overinterpret, would enhance the quality of manuscript.

Reviewer #2: This is an excellent study. See attacjed file for the following 2 comments:

I would change in the title the wording to "severe ENL".

I have one question. Whereas for severe ENL (post-MDT) the recommended treatment is prednisolone plus clofazimine, this is not taken as the reference treatment. Perhaps the authors ought to explain why.

Reviewer #3: This important multicentre RCT, conducted across several leprosy-endemic countries, evaluates the efficacy and safety of MTX as an adjunct to prednisone for the treatment of severe ENL. The study has been carefully designed, and the methods and analyses are described clearly and rigorously. The principal limitation of the trial, which the authors have acknowledged and discussed transparently, is that only 137 of the planned 416 participants were enrolled, representing 33% of the target sample size. Given the potential implications for statistical power and interpretation of the findings, this substantial shortfall should be stated more prominently in the Results section (include percentage).

Although the study is presented as a multinational trial, the vast majority of participants (85%) were recruited in India, with only small numbers enrolled in Ethiopia (4%), Indonesia (4), and Nepal (8%). This imbalance is unlikely to undermine the internal validity of the treatment comparison itself, assuming randomisation was maintained across sites. However, it does mean that the findings largely reflect the India healthcare system and patients, and as a result, impacts the generalisability of the results to other leprosy-endemic settings. Were any differences seen in primary and secondary outcomes between India and the other 3 countries? I would like to suggest that the authors acknowledge more explicitly in the Discussion that the study population was heavily weighted towards a single country and briefly discuss the implications for interpretation and broader applicability of the findings, particularly as in a previous French study with a small sample size a positive signal of MTX was observed (Jaume, PLoS Negl Trop Dis 2023).

Given initial high dosis of prednisone (40mg which will include multiple pills) and relative high number of pills for MTX/placebo, especially for the >60kg group receiving 20mg MTX/placebo, was pill burden for patients any concern?

Based on available data, any reason to further study MTX in ENL? Please elaborate and also include in the paper a brief discussion.

**********

PLOS authors have the option to publish the peer review history of their article (what does this mean?). If published, this will include your full peer review and any attached files.

If you choose “no”, your identity will remain anonymous but your review may still be made public.

**Do you want your identity to be public for this peer review?** For information about this choice, including consent withdrawal, please see our Privacy Policy.

Reviewer #1: No

Reviewer #2: No

Reviewer #3: No

[NOTE: If reviewer comments were submitted as an attachment file, they will be attached to this email and accessible via the submission site. Please log into your account, locate the manuscript record, and check for the action link "View Attachments". If this link does not appear, there are no attachment files.]

**Figure resubmission:**

**Reproducibility:**

To enhance the reproducibility of your results, we recommend that authors of applicable studies deposit laboratory protocols in protocols.io, where a protocol can be assigned its own identifier (DOI) such that it can be cited independently in the future. Additionally, PLOS ONE offers an option to publish peer-reviewed clinical study protocols. Read more information on sharing protocols at https://plos.org/protocols?utm_medium=editorial-email&utm_source=authorletters&utm_campaign=protocols

Attachment Submitted filename: PNTD-D-26-00496_reviewer 2.pdf

# Author comment: Author response to Decision Letter 1

**DOI:** 10.1371/journal.pntd.0014739.r003

5 Aug 2026

Attachment Submitted filename: PNTD-D-26-00496_response_letter.docx

# Peer review: Decision Letter 1

**DOI:** 10.1371/journal.pntd.0014739.r004

**Authors:** Epco Hasker, Ana LTO Nascimento

9 Sep 2026

Dear Dr. Barros,

We are pleased to inform you that your manuscript 'Methotrexate and Prednisolone compared to placebo and prednisolone in the treatment of severe Erythema Nodosum Leprosum - an international multicentre, double-blind randomised controlled clinical trial - MaPs in ENL' has been provisionally accepted for publication in PLOS Neglected Tropical Diseases.

Before your manuscript can be formally accepted you will need to complete some formatting changes, which you will receive in a follow up email. A member of our team will be in touch with a set of requests.

Please note that your manuscript will not be scheduled for publication until you have made the required changes, so a swift response is appreciated.

IMPORTANT: The editorial review process is now complete. PLOS will only permit corrections to spelling, formatting or significant scientific errors from this point onwards. Requests for major changes, or any which affect the scientific understanding of your work, will cause delays to the publication date of your manuscript.

Should you, your institution's press office or the journal office choose to press release your paper, you will automatically be opted out of early publication. We ask that you notify us now if you or your institution is planning to press release the article. All press must be co-ordinated with PLOS.

Thank you again for supporting Open Access publishing; we are looking forward to publishing your work in PLOS Neglected Tropical Diseases.

Best regards,

Epco Hasker

Academic Editor

PLOS Neglected Tropical Diseases

Ana LTO Nascimento

Section Editor

PLOS Neglected Tropical Diseases

Shaden Kamhawi

co-Editor-in-Chief

PLOS Neglected Tropical Diseases

orcid.org/0000-0003-4304-636XX

Paul Brindley

co-Editor-in-Chief

PLOS Neglected Tropical Diseases

orcid.org/0000-0003-1765-0002

***********************************************************

p.p1 {margin: 0.0px 0.0px 0.0px 0.0px; line-height: 16.0px; font: 14.0px Arial; color: #323333; -webkit-text-stroke: #323333}span.s1 {font-kerning: none

Reviewer's Responses to Questions

**Key Review Criteria Required for Acceptance?**

As you describe the new analyses required for acceptance, please consider the following:

**Methods**

-Are the objectives of the study clearly articulated with a clear testable hypothesis stated?

-Is the study design appropriate to address the stated objectives?

-Is the population clearly described and appropriate for the hypothesis being tested?

-Is the sample size sufficient to ensure adequate power to address the hypothesis being tested?

-Were correct statistical analysis used to support conclusions?

-Are there concerns about ethical or regulatory requirements being met?

Reviewer #1: (No Response)

Reviewer #3: -

**********

**Results**

-Does the analysis presented match the analysis plan?

-Are the results clearly and completely presented?

-Are the figures (Tables, Images) of sufficient quality for clarity?

Reviewer #1: (No Response)

Reviewer #3: -

**********

**Conclusions**

-Are the conclusions supported by the data presented?

-Are the limitations of analysis clearly described?

-Do the authors discuss how these data can be helpful to advance our understanding of the topic under study?

-Is public health relevance addressed?

Reviewer #1: (No Response)

Reviewer #3: -

**********

**Editorial and Data Presentation Modifications?**

Use this section for editorial suggestions as well as relatively minor modifications of existing data that would enhance clarity. If the only modifications needed are minor and/or editorial, you may wish to recommend “Minor Revision” or “Accept”.

Reviewer #1: (No Response)

Reviewer #3: -

**********

**Summary and General Comments**

Use this section to provide overall comments, discuss strengths/weaknesses of the study, novelty, significance, general execution and scholarship. You may also include additional comments for the author, including concerns about dual publication, research ethics, or publication ethics. If requesting major revision, please articulate the new experiments that are needed.

Reviewer #1: (No Response)

Reviewer #3: No further comments. Thank you for your diligent response to the Reviewers comments.

**********

PLOS authors have the option to publish the peer review history of their article (what does this mean?). If published, this will include your full peer review and any attached files.

If you choose “no”, your identity will remain anonymous but your review may still be made public.

**Do you want your identity to be public for this peer review?** For information about this choice, including consent withdrawal, please see our Privacy Policy.

Reviewer #1: No

Reviewer #3: No

# Editor report: Acceptance letter

**DOI:** 10.1371/journal.pntd.0014739.r005

**Authors:** Epco Hasker, Ana LTO Nascimento

Dear Dr. de Barros,

We are delighted to inform you that your manuscript, "Methotrexate and Prednisolone compared to placebo and prednisolone in the treatment of severe Erythema Nodosum Leprosum - an international multicentre, double-blind randomised controlled clinical trial - MaPs in ENL," has been formally accepted for publication in PLOS Neglected Tropical Diseases.

We have now passed your article onto the PLOS Production Department who will complete the rest of the publication process. All authors will receive a confirmation email upon publication.

The corresponding author will soon be receiving a typeset proof for review, to ensure errors have not been introduced during production. Please review the PDF proof of your manuscript carefully, as this is the last chance to correct any scientific or type-setting errors. Please note that major changes, or those which affect the scientific understanding of the work, will likely cause delays to the publication date of your manuscript. Note: Proofs for Front Matter articles (Editorial, Viewpoint, Symposium, Review, etc...) are generated on a different schedule and may not be made available as quickly.

Soon after your final files are uploaded, the early version of your manuscript will be published online unless you opted out of this process. The date of the early version will be your article's publication date. The final article will be published to the same URL, and all versions of the paper will be accessible to readers.

For Research Articles, you will receive an invoice from PLOS for your publication fee after your manuscript has reached the completed accept phase. If you receive an email requesting payment before acceptance or for any other service, this may be a phishing scheme. Learn how to identify phishing emails and protect your accounts at https://explore.plos.org/phishing.

Thank you again for supporting open-access publishing; we are looking forward to publishing your work in PLOS Neglected Tropical Diseases.

Best regards,

Shaden Kamhawi

co-Editor-in-Chief

PLOS Neglected Tropical Diseases

Paul Brindley

co-Editor-in-Chief

PLOS Neglected Tropical Diseases
